# Expansion of Human NK Cells Using K562 Cells Expressing OX40 Ligand and Short Exposure to IL-21

**DOI:** 10.3389/fimmu.2019.00879

**Published:** 2019-04-24

**Authors:** SoonHo Kweon, Minh-Trang Thi Phan, Sejong Chun, HongBi Yu, Jinho Kim, Seokho Kim, Jaemin Lee, Alaa Kassim Ali, Seung-Hwan Lee, Sang-Ki Kim, Junsang Doh, Duck Cho

**Affiliations:** ^1^School of Interdisciplinary Bioscience and Bioengineering (I-Bio), POSTECH, Pohang, South Korea; ^2^Department of Mechanical Engineering, POSTECH, Pohang, South Korea; ^3^Department of Laboratory Medicine, Chonnam National University, GwangJu, South Korea; ^4^Department of Health Sciences and Technology, SAIHST, Sungkyunkwan University, Seoul, South Korea; ^5^Immunotherapy Research Center, Korea Research Institute of Bioscience and Biotechnology, Daejeon, South Korea; ^6^Department of Biochemistry, Microbiology and Immunology, Faculty of Medicine, University of Ottawa, Ottawa, ON, Canada; ^7^Laboratory Animal Science, Department of Companion, Kongju National University, Yesan, South Korea; ^8^Department of Materials Science and Engineering, Seoul National University, Seoul, South Korea; ^9^Department of Laboratory Medicine and Genetics, Samsung Medical Center, Sungkyunkwan University School of Medicine, Seoul, South Korea; ^10^Stem Cell and Regenerative Medicine Institute, Samsung Medical Center, Seoul, South Korea; ^11^Department of Health Sciences and Technology, Samsung Advanced Institute for Health Sciences and Technology, Sungkyunkwan University, Seoul, South Korea

**Keywords:** natural killer cells, expansion, IL-21, K562, OX40 ligand

## Abstract

**Background:** Natural Killer (NK) cell-based immunotherapy used to treat cancer requires the adoptive transfer of a large number of activated NK cells. Here, we report a new effective method to expand human NK cells *ex vivo* using K562 cells genetically engineered (GE) to express OX40 ligand (K562-OX40L) in combination with a short exposure to soluble IL-21. In addition, we describe a possible mechanism of the NK cell expansion through the OX40 receptor-OX40 ligand axis which is dependent on NK cell homotypic interaction.

**Methods:** K562-OX40L cells were generated by lentiviral transduction and were used as feeder cells to expand and activate NK cells from PBMCs in the presence of IL-2/IL-15. Soluble IL-21 was also added in various concentrations only once at the beginning of the culture. NK cells were expanded for 4–5 weeks, and the purity, expansion rate, phenotype and function (cytotoxicity, antibody-dependent cell-mediated cytotoxicity (ADCC), cytokine production, CD107a degranulation) of these expanded NK cells were compared to those generated by using K562 feeder cells.

**Results:** The culture of NK cells with K562-OX40L cells in combination with the transient exposure to IL-21 highly enhanced NK cell expansion to approximately 2,000-fold after 4 weeks of culture, compared to a 303-fold expansion using the conventional K562 cells. Mechanistically, the OX40-OX40L axis between the feeder cells and NK cells as well as the homotypic interaction between NK cells through the OX40-OX40L axis were both necessary for NK cell expansion. The short exposure of NK cells to IL-21 had a synergistic effect with OX40 signaling for NK cell expansion. Apart from their enhanced expansion, NK cells grown with K562-OX40L feeder cells were similar to those grown with conventional K562 cells in regard to the surface expression of various receptors, cytotoxicity, ADCC, cytokine secretion, and CD107 degranulation.

**Conclusion:** Our data suggest that OX40 ligand is a potent co-stimulant for the robust expansion of human NK cells and the homotypic NK cell interactions through the OX40-OX40L axis is a mechanism of NK cell expansion.

## Introduction

The adoptive transfer of natural killer (NK) cells is a promising adjuvant approach for cancer immunotherapy, therefore obtaining sufficient numbers of activated NK cells is important for an effective NK cell-based immunotherapy. Several soluble cytokines such as IL-2 and IL-15 have been used to expand NK cells, but IL-2 alone or in combination with IL-15 results in minimal NK cell expansion (1). Other co-factors or stimuli (i.e., feeder cells) greatly enhanced the *ex vivo* expansion of NK cells. A higher fold expansion of NK cells was reported when both K562 cells and IL-2 were used, compared to IL-2 alone (2, 3). Recently, a remarkable activation and expansion of NK cells was achieved using K562 cells genetically engineered (GE) to express cytokines and co-stimulatory factors such as membrane-bound (mb) IL-15, mb IL-21, and 4-1BB ligand (4–6). Although 4-1BB ligand proved to be a key factor, additional novel co-stimulatory factors for NK cell activation and expansion are continuously being sought. In addition, the mechanism of NK cell expansion through the interaction between GE feeder cells expressing co-stimulatory factors and NK cells has not been elucidated.

A recent report demonstrated that stimulation of NK cells through the OX40 receptor increased NK cell IFN-γ production, cytotoxicity, and proliferation (7). In addition, OX40L was shown to be upregulated on NK cells stimulated with RPMI8866 or K562-mb15-41BBL feeder cells (8). Based on these results, we reasoned that OX40L would be a good candidate as a co-stimulatory factor to enhance human NK cell expansion, and produced a GE-K562 expressing OX40 ligand as feeder cells.

In this study, we evaluated the effects of expressing OX40L on K562 and the short exposure to IL-21 on *ex vivo* NK cell expansion by comparing conventional K562 and K562-OX40L based culture methods. In addition, we also studied a possible mechanism of NK cell expansion through the OX40-OX40L axis as well as the NK cell-NK cell homotypic interaction.

## Materials and Methods

### Cells and Culture

K562 (human myelogenous leukemia cell line) and Raji (human Burkitt's lymphoma cell line) were obtained from the American Type Culture Collection (ATCC, Manassas, VA, USA). The cells were cultured in RPMI 1640 medium supplemented with 10% heat-inactivated fetal bovine serum (FBS) (Gibco, US), 100 units/mL penicillin, and 100 μg/mL streptomycin (Invitrogen, CA, USA) at 37°C in a humidified 5% CO_2_ incubator.

### Generation of Genetically Engineered K562 Expressing OX40L

OX40L cDNA (accession number NM_003326) was cloned into the HIV-1 based, lentiviral expression vector that also encodes GFP (pLVX-IRES-ZsGreen1 from Clontech). The lentivirus was produced by a cotransfection with the psPAX2 packaging plasmid and the pMD2.G envelope plasmid. Recombinant lentivirus was harvested 72 h following cotransfection of the three vectors into 293T cells cultured in DMEM medium supplemented with 10% FBS. The transfections were performed using a lipofectamine 3000 (Invitrogen, CA) according to the manufacturer's instructions. The virus supernatant was purified, and the viral titer was determined. The K562 cells were seeded into 6-well plates at 5 × 10^5^ cells/well and incubated in 3 ml growth medium for 24 h before infection. The viral particles were added to the wells with 8 μg/ml polybrene. After a 24 h incubation at 37°C in 5% CO_2_, the virus-containing medium was removed and replaced with 3 ml of fresh culture medium. The infection was repeated three times every 48 h. The transduction efficiency was evaluated under an inverted fluorescence microscope on a daily basis. Two weeks after transduction, GFP-positive cells were sorted using BD FACSAria^TM^ III and then maintained in RPMI 1640 with 10% FBS.

### Cytokines and Antibodies

Recombinant human IL-2 and IL-15 (PeproTech, Rocky Hill, NJ, USA) were used to expand NK cells, and IL-21 (PeproTech) was used to stimulate NK cells at the beginning of the culture at various concentrations. Phycoerythrin (PE)-conjugated anti-human CD134 antibody (OX40 Ab, clone ACT35), and PE-conjugated anti-human CD252 antibody (OX40 Ligand Ab, clone ik-1) (BD Biosciences, San Jose, CA, USA) were used to measure the expression of OX40 receptor and its ligand on K562 and expanded NK cells during the culture. APC-Cy7-conjugated anti-human CD3, PE-Cy7-conjugated anti-human CD56, pacific blue-conjugated anti-human CD16, pacific blue-conjugated anti-human NKp46, PerCP-conjugated anti-human NKG2D, PerCP-conjugated anti-human CD8a, FITC-conjugated anti-human CD62L, FITC-conjugated anti-human CD57 (eBioscience, San Diego, CA, USA), FITC-conjugated anti-human CD3, APC-conjugated anti-human CD25, PE-conjugated anti-human CD69, APC-conjugated anti-human DNAM-1, APC-conjugated anti-human NKG2A, PE-conjugated anti-human NKG2C, PE-conjugated anti-human NKp30 (BD Biosciences) were used to evaluate the purity and surface expression of NK cell receptors. BV421-conjugated anti-human IFN-γ, APC-conjugated anti-human TNF-α (BD Biosciences) were used for intracellular staining, and PE-conjugated anti-human CD107a (BD Biosciences) was used as a surrogate marker of degranulation.

### Isolation of Human Peripheral Blood Mononuclear Cells and *ex vivo* Expansion of NK Cells

Our Institutional Review Board (No. SMC 2018-02-102) approved this study and no data were used for personal identification of human PBMCs. NK cells were expanded from peripheral blood mononuclear cells (PBMCs) by co-culture with irradiated conventional K562 or K562-OX40L cells as previously described, with a slight modification (4, 9). Briefly, human PBMCs were isolated from healthy adult donors using density-gradient centrifugation with Ficoll-Hypaque (*d* = 1.077, LymphoprepTM; Axis-Shield, Oslo, Norway) and washed twice with phosphate-buffered saline (PBS) (Welgene, USA). PBMCs were co-cultured with 100 Gy gamma ray-irradiated conventional K562 cells or K562-OX40L cells in a 24-well-plate with RPMI 1640 medium (10% FBS, 100 U/mL penicillin, 100 μg/mL streptomycin, and 4 mmol/L L-glutamine) containing 10 U/mL recombinant human IL-2. After 1 week, the concentration of IL-2 was increased to 100 U/mL and 5 ng/mL soluble IL-15 was added to the medium with irradiated conventional K562 or K562-OX40L feeder cells for re-stimulation at day 7, and day 14. The medium was replaced every 2–3 days. To optimize the protocol for NK cell expansion using our own genetically modified K562-OX40L feeder cells, soluble IL-21 was added at various concentration (0, 5, 50, 100 ng/ml) on day 0 of culture. Expanded NK cells were harvested on days 14 and 21, and used for further experiments. Cell expansion was presented as the “expansion fold,” which was calculated by dividing the absolute output number of NK cells after every 7 days of culture by the respective number on day 0. The absolute number of NK cells was calculated by multiplying the total viable number of cells by the percentage of CD56+CD3- cells determined by flow cytometry (10, 11).

### Surface and Intracellular Staining Using Flow Cytometry

The expression of OX40L on conventional K562 and K562-OX40L cells, as well as the expression of NK cell receptors on expanded NK cells were examined by flow cytometry during the first week of culture using an appropriate combination of fluorochrome-conjugated monoclonal antibodies (CD3, CD56, CD16, CD25, CD69, NKp46, NKG2D, DNAM-1, OX40L, OX40R). Briefly, 1 × 10^5^ K562, K562-OX40L, and expanded NK cells were washed with FACS buffer (PBS containing 1% FBS) and stained with the appropriate combination of fluorochrome-conjugated monoclonal antibodies for 30 min on ice in the dark. After washing with FACS buffer, the stained cells were acquired using a FACS Canto II (BD Biosciences). The data were analyzed using Kaluza software version 1.3 (Beckman Coulter, Brea, CA, USA). To examine the phenotypic characteristics of the expanded NK cells after 2 weeks in culture, the NK cells (1 × 10^5^) were washed with FACS buffer and then stained with an appropriate combination of fluorochrome-conjugated monoclonal antibodies (CD3, CD56, NKG2A, DNAM-1, NKG2D, CD8a, NKG2C, NKp30, CD57, CD62L, CD16, NKp46). For the measurement of IFN-γ and TNF-α production, intracellular staining using the BD Cytofix/Cytoperm™ kit (BD Biosciences) was performed according to the manufacturer's instructions. Briefly, the expanded NK cells (5 × 10^5^) were co-cultured with K562 cells (5 × 10^5^) in a 96-well U-bottom plate in the presence of brefeldin A at 37°C and 5% CO_2_ for 5 h. The cells were then harvested and washed with FACS buffer followed by staining with FITC-conjugated anti-human CD3 and PE-Cy7-conjugated anti-human CD56 for 15 min on ice. The NK cells were then washed, fixed, and permeabilized, before being stained with PE-conjugated anti-human IFN-γ and APC-conjugated anti-human TNF-α on ice for 30 min. Finally, the cells were washed, and analyzed using FACS Canto II and Kaluza software.

### CD107a Degranulation

To assess the degranulation of expanded NK cells against K562 target cells, 5 × 10^5^ expanded NK cells on day 21 were co-cultured with 5 × 10^5^ K562 cells for 1 h in a 96-well U-bottom plate in the presence of 5 μL/mL PE-conjugated anti-human CD107a. After 1 h, monensin and brefeldin A (BD Biosciences) were added and the plate was incubated for an additional 4 h. The NK cells were then washed and stained with FITC-conjugated anti-human CD3 and PE-Cy7-conjugated anti-human CD56 on ice for 15 min. The cells were washed with FACS buffer and analyzed using FACS Canto II and Kaluza software.

### RT-PCR Detection of OX40L mRNA

For the quantitation of mRNA levels that encode OX40L, RT-PCR was performed using K562, K562-OX40L, and NK cells which were isolated by NK cell enrichment cocktail (Stem cell Technologies) after 3 days of co-culture of PBMCs with feeder cells. Briefly, total RNA were isolated by High Pure RNA Isolation Kit (Roche) and cDNA were synthetized by RevertAid First Strand cDNA Synthesis Kit (Thermo scientific). These first-strand cDNA were amplified with a OX40L specific primer set (sense 5′-CTGCTCCTGTGCTTCACCTAC-3′ and anti-sense 5′-TCCAGGGAGGTATTGTCAGTG-3′) or a GAPDH specific primer set (sense 5′-AGCCACATCGCTCAGACAC-3′ and anti-sense 5′-GCCCAATACGACCAAATCC-3′) with thermal cycling conditions at 95°C for 15 min followed by 32 cycles of 95°C for 30 s, 53°C for 30 s, and 72°C for 90 s. The amplified products were run on a 2.5% agarose gel electrophoresis. Relative RT-PCR quantification was performed by measuring OX40L gene signal relative to GAPDH signal using Image J analysis software after color inversion of the image.

### Cytotoxicity and ADCC Assays

The cytotoxicity of expanded NK cells against target tumor cells (K562 and Raji cells) were measured by a flow cytometry-based NK cytotoxicity assay using Carboxyfluorescein Diacetate Succinimidyl Ester (CFSE) (Life Technologies) staining of target cells. Briefly, K562 target cells were stained with 0.5 μM CFSE in FACS buffer for 10 min at 37°C. To perform the ADCC assay, Raji cells were first incubated with 1 μg/ml of rituximab for 4 h and then stained with CFSE. Cells were then washed twice using complete media. CFSE-stained target cells (5 × 10^5^) were placed in a 96-well U-bottom plate in triplicate and then mixed with various numbers of expanded NK cells (0.5:1, 1:1, and 2:1 effector-to-target (E:T) ratios). The plates were centrifuged at 1,500 rpm for 3 min and then incubated for 4 h at 37°C in a 5% CO_2_ incubator. The mixed cells were transferred to FACS tubes after 4 h. Before acquisition (5–10 min), 1 μl of 1 mg/ml propidium iodide (PI) (SigmaAldrich, St. Louis, MO, USA) was added to each tube. The cells were acquired on a FACS Canto II and analyzed using Kaluza software. Percentages of dead target cells showing CFSE-positive and PI-positive were calculated after subtracting the percentage of spontaneous death of target cells.

### Evaluation of Remaining K562-OX40L Feeder Cells When Co-cultured With PBMCs

To check the survival of K562-OX40L feeder cells during NK cell expansion, γ-irradiated K562-OX40L were stained with 5 μM CFSE prior to co-culturing with PBMCs. CFSE-positive K562 and PI-stained dead cells were identified every day by flow cytometry.

### CFSE Dilution Assay

The CFSE dilution assay was performed as previously described, with a slight modification (12). Briefly, PBMCs were co-cultured with K562-OX40L for day 3 in the presence of 10 U/mL of IL-2 and NK cells were isolated and labeled with 5 μM CFSE. Cells were washed twice in PBS, and 0.1 × 10^5^ NK cells labeled with CFSE were incubated with 96 well-flate bottom or round-bottom plate with 100 U/mL IL-2 and 1 μg/ml of IgG or Oxelumab. Then NK cells were harvested every 2 days and assayed.

### OX40L Blocking Assay

To explore the role of OX40/OX40L axis in NK cell expansion, OX40L blocking Ab (oxelumab) or human IgG Ab were added at 10 μg/ml on days 3 of culture. NK cell purity and expansion fold were checked at days 7, 14, 21, 28 of culture.

### Statistics

Statistical analyses of the differences between the groups in regard to the purity, fold expansion, and cytotoxicity of expanded NK cells were carried out using the Mann-Whitney *U*-test, with a *p* ≤ 0.05 considered to indicate significance.

## Results

### Generation of Genetically Engineered K562 Expressing OX40L

**Figure 1:**
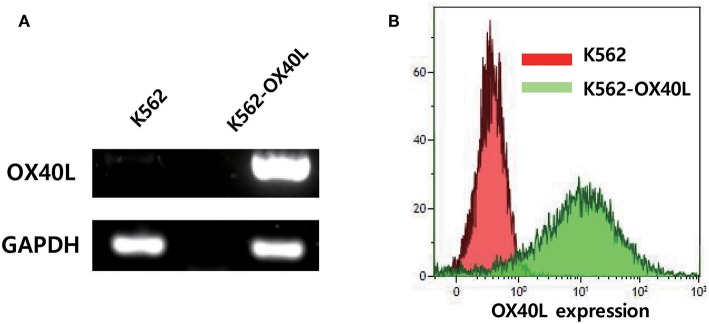
OX40L expression on genetically engineered K562. **(A)** OX40L mRNA expression in K562 and K562-OX40L was determined by RT-PCR with GAPDH serving as control **(B)** OX40L surface expression was analyzed by FACS using anti-OX40L (clone ik-1) in K562 (red) and K562-OX40L (green) cells.

To determine whether the overexpression of OX40L on K562 feeder cells would enhance NK cell expansion *ex vivo*, we generated a K562 cell line that expresses OX40L by using lentiviral transduction. The enhanced OX40 ligand expression on K562-OX40L cells was confirmed at the transcriptional and translational levels. Messenger RNA (mRNA) levels of the OX40 ligand were detected on the transduced cells, but not on parental K562 cells (Figure 1A). Consistently, flow cytometry analysis revealed that K562-OX40L cells had a high surface expression of OX40L, whereas the conventional K562 cells showed negligible expression of surface OX40L (Figure 1B). The expression of OX40L on K562-OX40L cells remained stable for at least 4 months.

### K562-OX40L Feeder Cells Improve the Expansion of NK Cells From PBMCs

**Figure 2:**
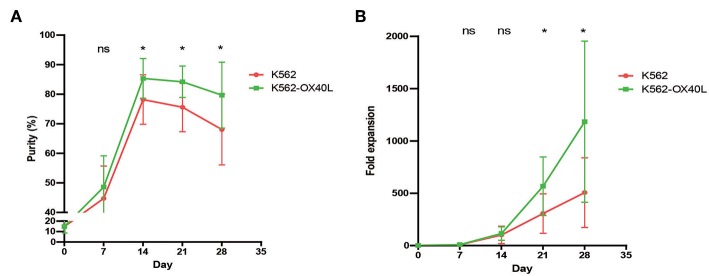
The purity and fold expansion of expanded human NK cells for 4 weeks. Freshly prepared PBMCs were co-cultured with γ-irradiated K562 (red) and K562-OX40L (green) feeder cells at day 0 in the presence of 10 U/mL of IL-2 and re-stimulated at day 7, 14 with 100 U/mL of IL-2 and 5 ng/mL of IL-15. The medium was replaced every 2–3 days. **(A)** The purity of expanded NK cells were determined by flow cytometry using FITC-conjugated anti-human CD3 and PE-Cy5-conjugated anti-human CD56. NK cell purities in two groups were significantly different from day 14 of culture. **(B)** Fold expansion of NK cells in the K562 vs. K562-OX40L added groups were significantly different from day 21 of culture. [K562, *n* = 7; and K562-OX40L, *n* = 8; error bars, mean ± standard deviation (SD); ns, not significant; *p < 0.05].

To test whether K562-OX40L cells promote NK cell expansion, we compared the purity and fold expansion of NK cells after culture with either K562 or K562-OX40L feeder cells. An increased purity of expanded NK cells was observed when grown with K562-OX40L cells after 2 weeks of culture (Figure 2A). Consistently, and the fold expansion of NK cells in cultured with K562-OX40L cells was significantly higher than that of NK cells cultured with K562 cells from 3 to 4 weeks of culture (Figure 2B). Median (range) was 235 (125.8 – 594.6), 303 (66.7 – 1261.3) in the K562 group; 486.3 (261.4 – 1096.7), 1013.5 (578.9 – 2959.5) in K562-OX40L group, respectively.

### Characteristics of Expanded NK Cells Were Similar in the Two Groups

**Figure 3:**
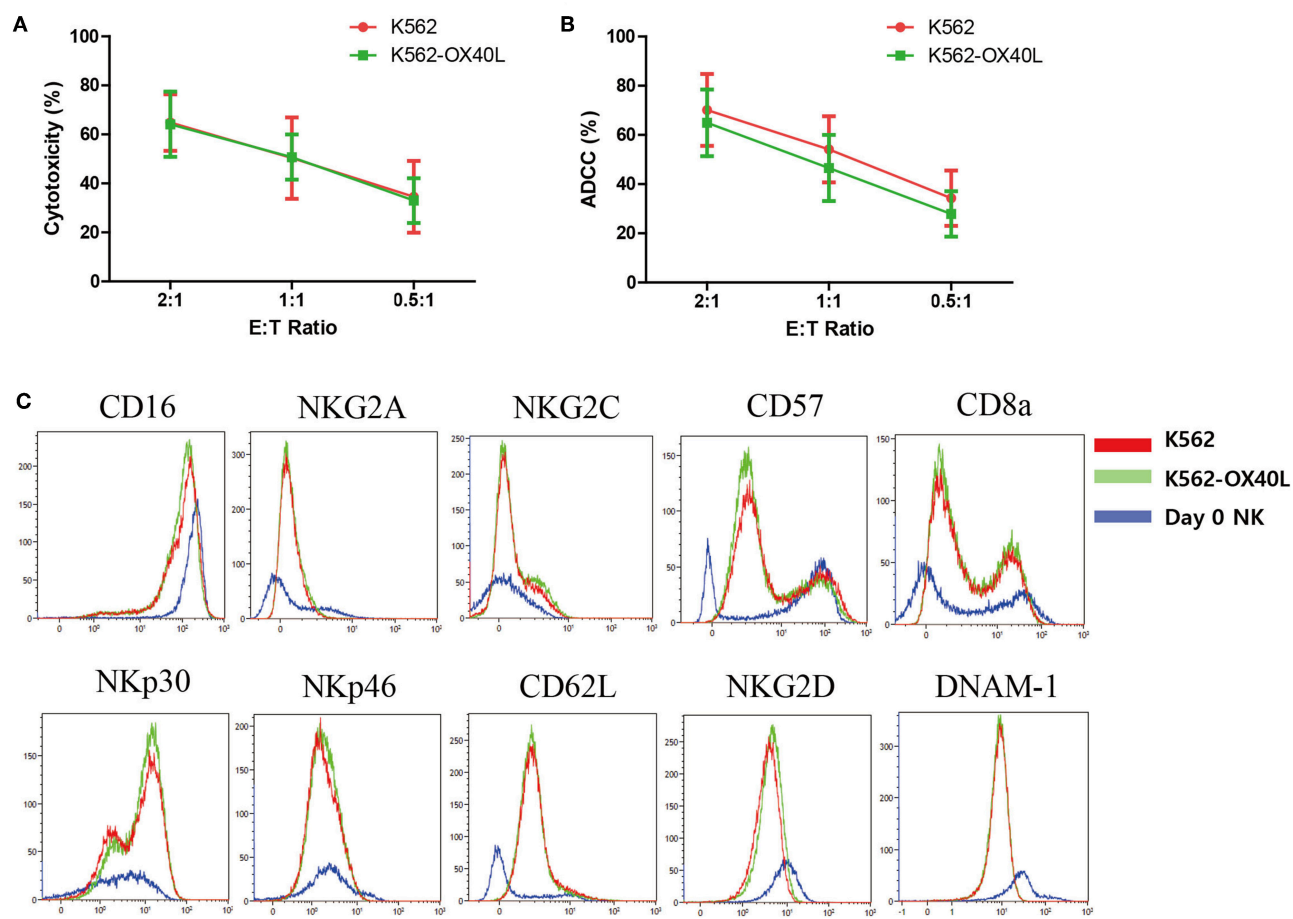
Characteristics of expanded NK cells on day 14 in K562 and K562-OX40L group. **(A)** The cytotoxicity of expanded NK cells toward K562 cells and **(B)** ADCC of expanded NK cells against rituximab-coated Raji cells were measured by CFSE-based flow cytometry assay at effector-to-target (E:T) ratio of 2:1, 1:1, 0.5:1 for 4 h. Results presented are the mean of 6 donors and error bars represent the mean ± SD. **(C)** Expression of surface receptors on NK cells were assayed for CD16, NKG2A, NKG2C, CD57, CD8a, NKp30, NKp46, CD62L, NKG2D, and DNAM-1. Representative plots are shown in NK cell subset from the same donor, K562 group (red), and K562-OX40L group (green), NK cells on day 0 before co-culture (blue).

To compare the cytotoxicity of NK cells expanded with either K562 or K562-OX40L cells, we investigated the direct cytotoxicity (Figure 3A) and ADCC (Figure 3B) of expanded NK cells at day 14. There were no significant differences found between NK cells expanded with either K562 or K562-OX40L cells at various E:T ratios. The expression of various surface receptors (CD16, NKG2A, NKG2C, CD57, CD8a, NKp30, NKp46, CD62L, NKG2D, and DNAM-1) were similar on NK cells expanded at day 7 and 14 with either K562 or K562-OX40L feeder cells (Figure 3C and Figure S1 in Supplementary Material).

### OX40L Stimulation and Short Exposure to IL-21 Result in a Remarkable Expansion of NK Cells

**Figure 4:**
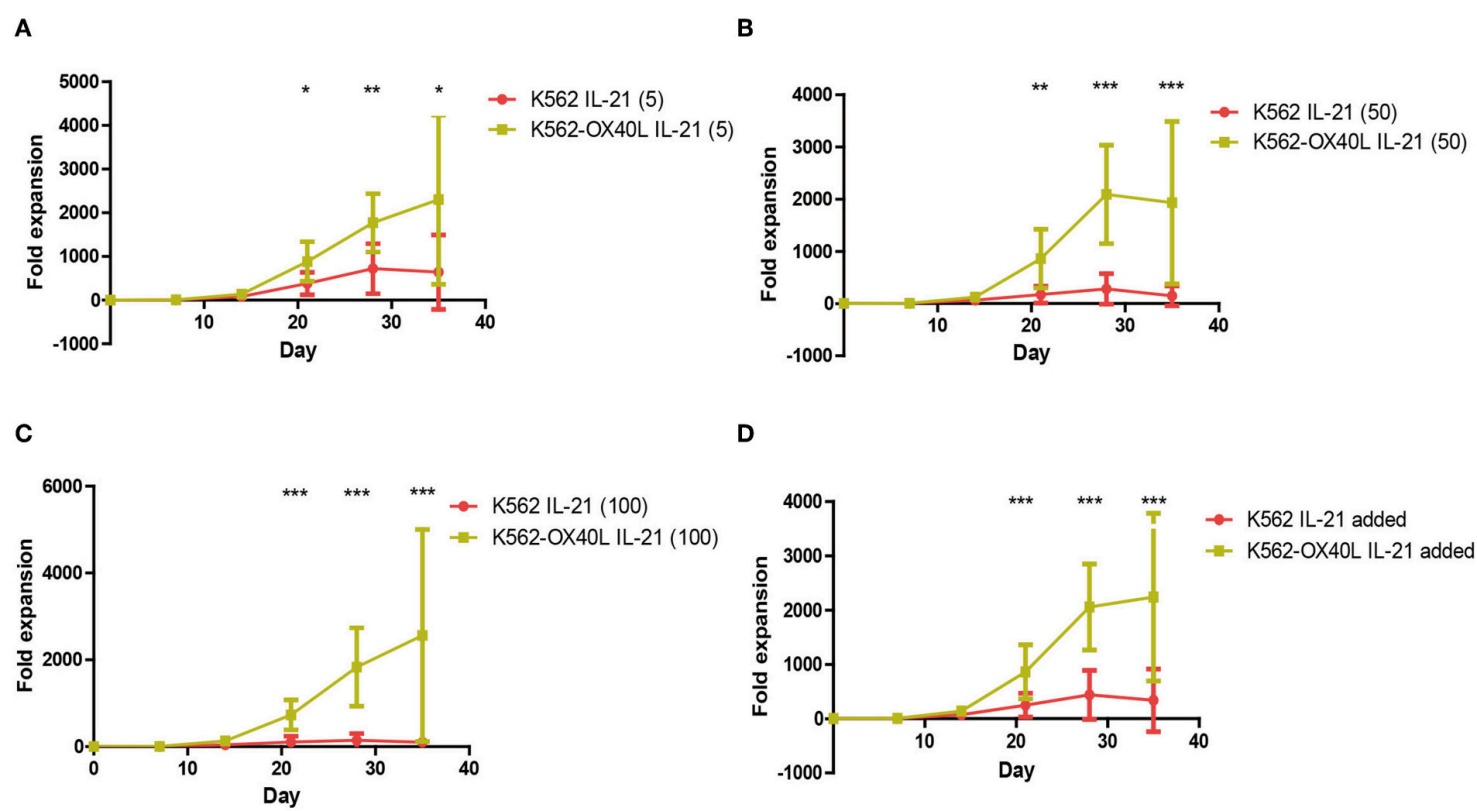
Irradiated K562-OX40L with short exposure to different concentration of IL-21 results in pronounced expansion of NK cells for 5 weeks. PBMCs were co-cultured with γ-irradiated K562 (red) or K562-OX40L (green) with 10 U/mL of IL2 and 5 ng/ml **(A)**, 50 ng/ml **(B)**, 100 ng/ml **(C)** of IL-21 at day 0 and re-stimulated at day 7, 14 with 100 U/mL of IL-2 and 5 ng/mL of IL-15. The medium was replaced every 2–3 days. **(D)** Each of IL-21 added groups was combined and compared within K562 and K562-OX40L groups (K562, *n* = 7; and K562-OX40L, *n* = 10; error bars, mean ± SD; *p < 0.05; **p < 0.01; ***p < 0.001).

IL-21 is one of the known cytokines that augment NK cell expansion *ex vivo*. To improve the purity and fold expansion of NK cells cultured with K562-OX40L, a short exposure to IL-21 was added to the established culture condition (13, 14). The culture condition using a combination of short exposure to IL-21 and K562-OX40L showed significantly higher fold expansion at all concentrations of IL-21 used, compared to conditions in the absence of IL-21. The maximum median (range) fold expansions of NK cells at various concentrations of IL-21 (5, 50, 100 ng/mL) were 1809.2 (506.6 – 6074.3), 1384.7 (330.6 – 5219.3), 1987.5 (301.3 – 7093.7) at day 35, respectively (Figure 4). In contrast to the combination of a short exposure to IL-21 and K562-OX40L cells, the combination of a short exposure to IL-21 (50, 100 ng/ml) and K562 cells showed a negative effect on NK expansion.

### Characteristics of NK Cells Expanded With Short Exposure to IL-21 and K562-OX40L Cells

**Figure 5:**
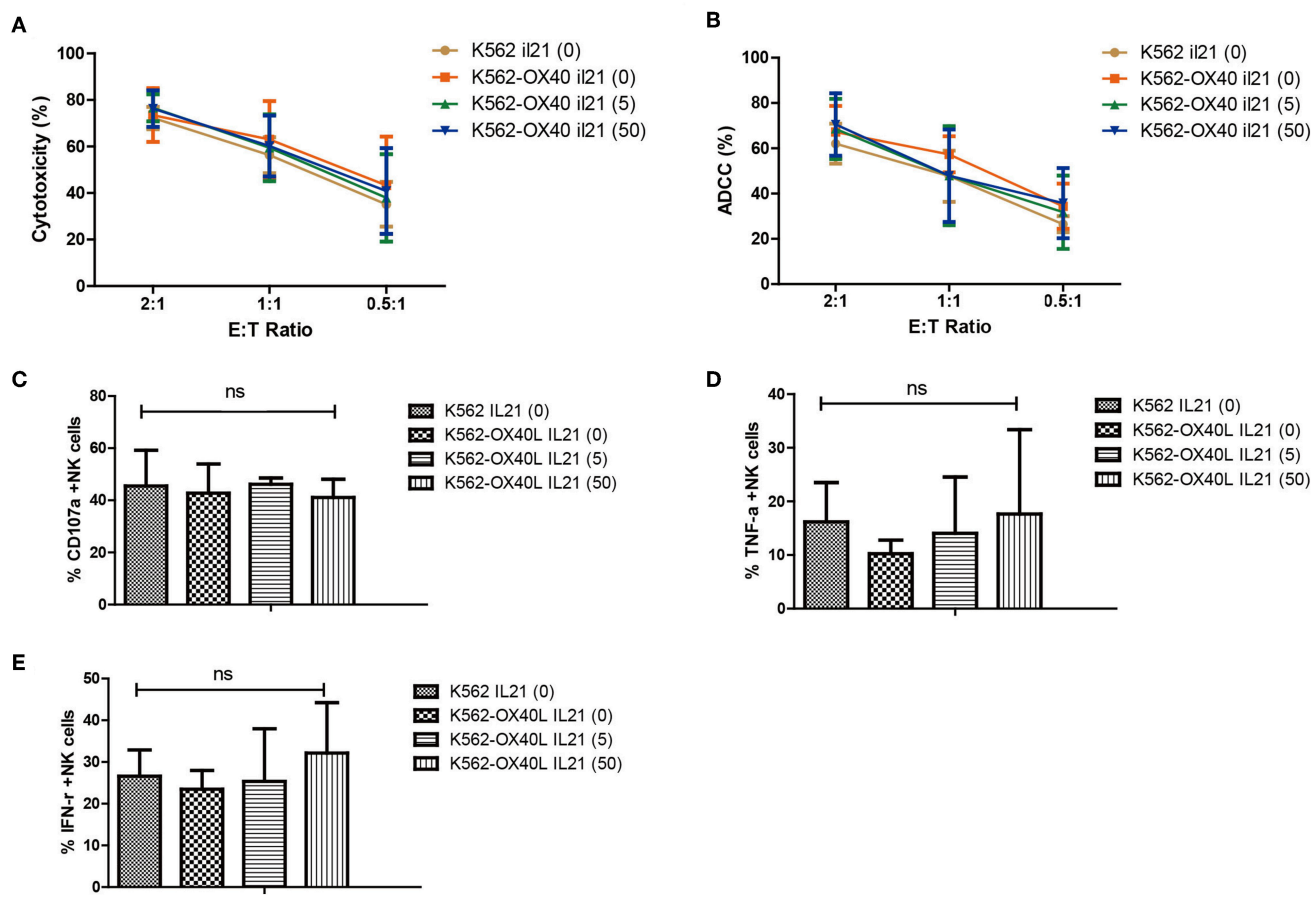
Characteristics of expanded NK cells in K562, K562-OX40L groups without IL-21 and various concentration of IL-21 (5.50 ng/ml) added K562-OX40L groups on day 21. Direct cytotoxicity of expanded NK cells toward K562 cells **(A)** and ADCC of rituximab-coated Raji cells **(B)** were measured at E:T ratio of 2:1, 1:1, 0.5:1 for 4 h using CFSE-based flow cytometry assay. The results presented are means ± SD samples from the four donors. Surface expression of CD107a **(C)** and intracellular expression of TNF-α **(D)** and IFN-γ **(E)** of expanded NK cells was measured by incubating expanded NK cells with K562 cells at an effector-to-target (E:T) ratio of 1:1 for 5 h and evaluated by means of flow cytometry. Bar graphs show the percentage of expanded NK cells for degranulation (CD107a) and production of TNF-α and IFN-γ for the four groups. (error bars, mean ± SD; ns, not significant).

The phenotypic characteristics of NK cells expanded with either K562 or K562-OX40L cells in the presence or absence of IL-21 were compared at day 21. Similar cytotoxicity (Figure 5A), ADCC (Figure 5B), percentage of CD107a (degranulation assay) (Figure 5C), TNF-α -positive cells (Figure 5D), and IFN-γ (Figure 5E) were observed regardless of IL-21 addition.

### Expression of OX40L Was Highly Induced on NK Cells During Expansion With K562-OX40L

**Figure 6:**
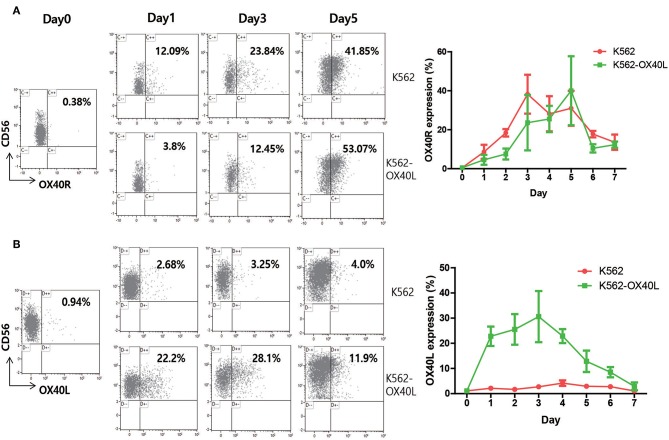
OX40R and OX40L expression on human NK cells. PBMCs of healthy donors were co-cultured with either K562 or K562-OX40L feeder cells. Percentage of surface expression of OX40R **(A)** and OX40L **(B)** on NK cells were analyzed on a daily basis using APC-Cy7-conjugated anti-human CD3, PE-Cy7-conjugated anti-human CD56 and Phycoerythrin (PE)-conjugated anti-human CD134 (OX40 Ab, clone ACT35) and PE-conjugated anti-human CD252 (OX40 Ligand, clone ik-1) for 1 week. (K562, *n* = 4; and K562-OX40L, *n* = 4; error bars, mean ± SD).

To dissect the mechanism by which OX40/OX40L interaction enhances NK cell expansion *ex vivo*, the surface expression of both OX40 and OX40L and other receptors on NK cells was analyzed every day for 1 week (Figures 6A,B and Figure S2 in Supplementary Material). Although the expression of both OX40 and OX40L on resting NK cells among PBMCs was not observed, the expression of OX40 on expanding NK cells was increased with either K562 or K562-OX40L feeder cells, indicating that OX40 expression was induced by NK cell activation (15). Interestingly, the expression of OX40L was highly induced on NK cells during expansion with K562-OX40L, but not with K562 feeder cells (Figure 6B).

### OX40L On NK Cells Is Generated After Transient Exposure to K562-OX40L Cells and Is Then Maintained Only Through NK Cell-NK Cell Interaction, Irrespective of K562-OX40L Feeder Cells

**Figure 7:**
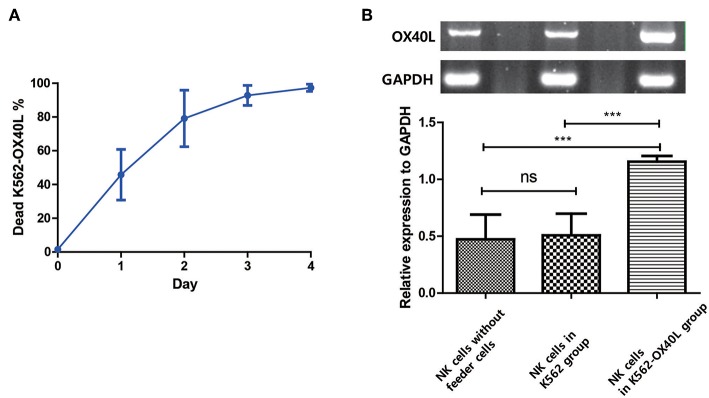
OX40L on NK cells is generated after exposure to K562-OX40L and maintained during NK cell expansion **(A)** Survival of K562-OX40L feeder cells during NK cell expansion. γ-irradiated K562-OX40L were stained with 5μM CFSE prior to co-culturing with PBMCs. Then, CFSE-positive K562 and PI-stained dead cells were identified every day by flow cytometry. *n* = 3 error bars, mean ± SD **(B)** OX40L mRNA expression in NK cells. NK cells were isolated by using NK cell enrichment kit at day 3 from PBMCs without feeder cells, PBMCs with either K562 or K562-OX40L culture conditions. Then, RT-PCR with *GAPDH* serving as control was performed. Band intensity from RT-PCR results were quantified using Image J after color inversion of the image. OX40L gene expression relative to GAPDH expression was analyzed from triplicate samples of each group. (*n* = 3 error bars, mean ± SD; ***p < 0.001).

To determine how OX40L on NK cells is generated and maintained during NK cell expansion, the viability of K562-OX40L feeder cells and OX40L mRNA in isolated NK cells were measured by flow cytometry and RT-PCR, respectively. Approximately 90% of K562-OX40L feeder cells were dead at day 3 (Figure 7A), suggesting that the OX40-OX40L interaction between NK cells and K562-OX40L is restricted to 3 days. To assess whether the interactions between NK cells and K562-OX40L cells induced the synthesis of mRNA encoding OX40L, NK cells isolated by using NK cell enrichment kit from expansion cultures (PBMCs without feeder cells, PBMCs with either K562 or K562-OX40L) at day 3 were analyzed by RT-PCR. Although the mRNA levels of OX40L were detected in the condition without K562-OX40L, NK cells co-cultured with K562-OX40L showed a higher mRNA expression of OX40L with significant differences to other groups (Figure 7B), consistent with a high surface expression of OX40L as measured by flow cytometry.

### OX40-OX40L Axis Is Associated With the Expansion of NK Cells

**Figure 8:**
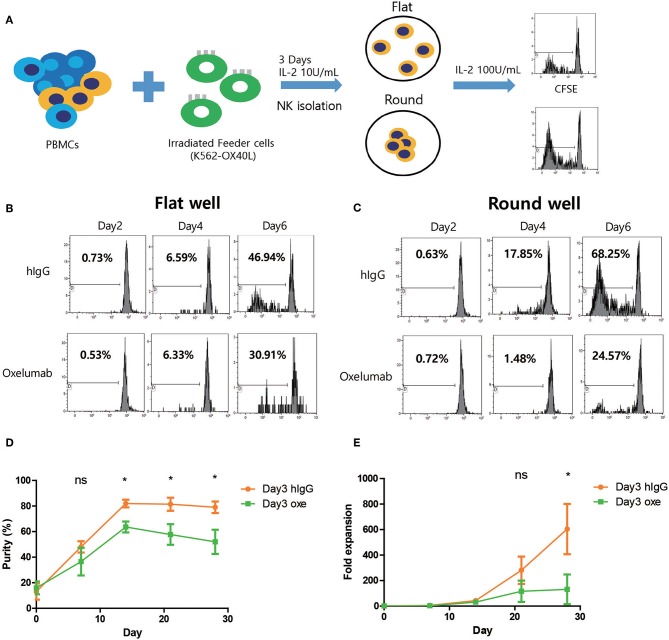
Blocking of OX40-OX40L axis inhibits NK cell proliferation. **(A)** Freshly prepared PBMCs were co-cultured with γ-irradiated K562-OX40L feeder cells in the presence of 10 U/mL of IL-2 for 3 days and NK cells were isolated to 0.1 × 10^5^ NK cells/well with 100 U/mL of IL-2 and IgG or Oxelumab in flat-bottom or round-bottom wells in 96-well plates. **(B,C)** CFSE dilution assay was performed every 2 days in flat or round-bottom wells. Data are representative of three independent experiments. **(D)** The purity and **(E)** fold expansion of expanded NK cells in human IgG (orange dots) and Oxelumab (green squares) treated group were measured. OX40L blocking antibodies (Oxelumab) were added at day 3 when OX40L expression was peaked. Purities and fold expansion in two groups significantly different from day 14 and day 21 of culture, respectively. (hIgG treated group, *n* = 3; and Oxelumab treated group, *n* = 5; error bars, mean ± SD; ns, not significant; *p < 0.05).

To investigate the role for homotypic interactions of OX40 and OX40L on NK expansion, NK cells co-cultured with K562-OX40L for 3 days were isolated and cultured in round or flat-bottom 96-well plates and CFSE dilution assay was performed (Figure 8A). Similar with previous study, NK cells in round-bottom wells with hIgG treatment exhibited higher levels of proliferation compared to those in flat-bottom wells (Figures 8B,C) and blocking of the OX40/OX40L interaction with OX40L blocking antibodies (Oxelumab) reduced proliferation levels of NK cells especially in round wells (Figures 8B,C) suggesting that NK cell-to-cell interactions through OX40-OX40L axis in NK cell proliferation is important. To further investigate whether OX40L expression on NK cells expanded using K562-OX40L feeder cells contributed to the enhanced expansion, Oxelumab or isotype control were added at day 3 of culture (peak day of OX40L expression). Notably, blockade of the OX40/OX40L interaction by Oxelumab significantly reduced the purity after day 14 (Figure 8D) and expansion folds of NK cells expanded in the presence of K562-OX40L feeder cells after day 28 (Figure 8E), demonstrating that OX40L expressed on the activated NK cells is necessary for enhanced NK cell expansion during culture with K562-OX40L feeder cells.

## Discussion

In this report, we developed a new *ex vivo* expansion method of human NK cells from PBMCs using irradiated K562 feeder cells expressing OX40 ligand and showed that a short exposure to IL-21 might have a synergistic effect with OX40 signaling for NK expansion. In addition, we describe a possible mechanism of NK cell expansion in regard to OX40 receptor- OX40 ligand based on NK cell homotypic interactions.

IL-2 alone or in combination with IL-15 is essential for NK cell expansion, but costimulatory signals are required for the optimal proliferation of NK cells. One method to provide costimulatory signals is to co-culture NK cells with irradiated feeder cells, which are assumed to contain costimulatory factors. Robertson et al. reported that stimulation with NK-sensitive K562 cells strongly augmented CD56^dim^ NK cell proliferation and suggested that NK cells require multiple signals for optimal proliferation (2).

Our group has tried to optimize the NK cell expansion protocol with conventional K562 feeder cells by using protocol described previously with slight modification (4). A low concentration of IL-2 (10 U/mL) was used to minimize proliferation of T cells in the first week and then increased to 100 U/mL of IL-2 and additionally 5 ng/mL of IL-15 was also used for the rest of the culture. In this study, we achieved 303 (66.7 – 1261.3) median (range) fold expansions of NK cells after day 28 from PBMCs by co-culturing with γ-irradiated K562 feeder cells in the presence of IL-2/IL-15. Similar protocol with K562 feeder cells was reported by Bae et al. However, they reported that expansion of NK cell from CD3^+^ depleted lymphocytes with IL-2 and mitomycin-C (MMC) treated K562 achieved 20-fold for 15 days (3). Presumably, our highly efficient NK expansion protocol might be due to difference ratio between PBMCs and K562 or different cytokine combination or using irradiated feeder cells rather than MMC treated cells.

Recently, remarkable proliferation of NK cells was achieved using GE-K562 cells expressing cytokines and co-stimulants [i.e., K562 expressing membrane-bound (mb) IL-15 and 4-1BBL, K562 expressing 4-BBL with mb IL-21] (4–6). For example, K562 expressing 4-BBL with mb IL-21 promoted log-phase NK cell expansion by 47,967-fold with increased telomere length and enhanced activation of the STAT-3 signaling pathway for sustained expansion of NK cells without senescence (5). In the study, GE-K562 cells expressing a co-stimulant factor was considered to develop a new effective method for the *ex vivo* expansion of human NK cells.

It appears that 4-1BB ligand is a key co-stimulant for the preferential expansion of human NK cells. 4-1BBL is a member of the TNFR/TNF ligand family, which are expressed on T cells and antigen-presenting cells (APCs), respectively (16, 17). OX40L, another member of the TNF superfamily, is also induced on APCs such as B cells, dendritic cells, and macrophages to control T cell priming following the recognition of antigen (18, 19). Additionally, several groups have shown that OX40L is also expressed on activated T cells and OX40-OX40 ligand interaction within T cells stimulate T cell activation and survival (20–22). Even though there is a lack of understanding of the role of OX40-OX40 ligand interaction in NK cells, recent studies proved that induction of OX40 on NK cells was dependent on close proximity and stimulation of OX40 increased IFN-γ production, cytotoxicity and proliferation (7).

Therefore, we selected OX40L as an alternative co-stimulant for the preferential expansion of human NK and developed a GE-K562 cell line expressing OX40L. When the efficiency of NK cells expansion was compared using the two types of feeder cell lines (K562 cells and K562-OX40L), the purity of NK cells cultured with K562-OX40L cells was significantly higher than that of NK cells cultured with K562 cells from days 14 to 28 with a final median fold expansion of 303 and 1013.5 at day 28, respectively. Although we did not compare NK cells' characteristics and fold expansion when using K562-OX40L vs. K562-mb15-41BBL/K562-mb IL-21 based methods, our results reveal that OX40L is a co-stimulant for the preferential expansion of human NK cells, and that NK cell proliferation might be induced by OX40/OX40L mediated cell-cell interaction.

Interestingly, the improvement of purity and fold expansion of NK cells grown with K562-OX40L was minimal and the characteristics of these NK cells were similar to that of NK cells grown with K562. Our group previously showed that a short exposure to IL-21 resulted in a strong proliferative response of NK cells when cultured with K562-mb15-41BBL cells. Although IL-21 has been known to induce apoptosis in activated NK cells that was cultured with IL-2/IL-15, the short pulse of IL-21 in combination with the IL-2 and IL-15 was able to prevent the induced apoptosis and resulted in increased NK cell expansion (13). Granzin et al. also reported that repeated stimulation with irradiated EBV-LCL and IL-2 and addition of IL-21 at the initiation of the culture allowed sustained NK cell proliferation with 10^11^-fold NK cell expansion after 6 weeks (14). Based on an idea that OX40L, like 41BBL, could synergize with IL-21 to enhance NK cell expansion, we applied the short exposure to soluble IL-21 by adding IL-21 once at the beginning of the culture in our NK cell expansion protocol using irradiated K562 or K562-OX40L cells. The effect of a short exposure to IL-21 on NK cell expansion was compared between K562 and K562-OX40L feeder cells. Notably, the modified protocol using K562 cells with a short exposure to IL-21 showed only minor effects on NK cell expansion, whereas using K562-OX40L cells with a short exposure to IL-21 showed highly enhanced NK expansion. Consistent with previous results (13, 14), this data implies that OX40L also has a synergistic effect with short exposure to IL-21 for NK cells expansion, similar to 41BBL. To further investigate this synergic effect, telomere length assay was performed (data not shown). However, in contrast to previous findings, K562-OX40L/IL-21-expanded NK cells did not show a significant increase in telomere length compared to K562/IL-21-expanded NK cells. Presumably, this finding might be due to our assay's limited sensitivity to detect subtle difference between two culture protocols. Unfortunately, we did not directly compare NK expansion using K562-OX40L based method with other genetically modified K562 cells (expressing membrane bound IL-15, IL-21, 4-1BBL) and it is a limitation of this study.

The characteristics of NK cells expanded using the modified protocol including a short exposure to IL-21 were compared to those NK cells by K562 based method, not IL-2/IL-15, in the absence of K562, based method. NK cells generated by the various concentrations of IL-21 in the presence of K562-OX40L or K562 cells showed comparable cytolytic activity, ADCC and CD107a, IFN-γ, and TNF-α expression. These results suggest that the function of NK cells depends on IL-2/IL-15 and K562 feeder cells rather than OX40L or a short exposure to IL-21.

To investigate whether OX40L was generated and expressed by NK cells after the interaction with K562-OX40L cells or whether OX40L was transferred from K562-OX40L cells by trogocytosis, the mRNA levels of OX40L and its surface expression were investigated by RT-PCR and flow cytometric analysis, respectively, using NK cells isolated by NK cell enrichment cocktail kit from cell culture at day 3. Although the mRNA encoding OX40L was detected in NK cells cultured with K562, and K562-OX40L or without feeder cells because of the relatively high sensitivity of RT-PCR, only NK cells in K562-OX40L group displayed substantial surface expression of OX40L. These findings indicate that the activation of NK cells with OX40 through OX40-OX40L axis is sufficient for the induction of OX40L expression on NK cells in line with previous findings where the upregulation of OX40L expression was dependent on 4-1BB stimulation (8).

A previous study revealed that homotypic interactions among activating NK cells enhances NK activation and proliferation (12). To investigate the role of homotypic OX40/OX40L interaction on NK expansion, we cultured isolated NK cells in round or flat-bottom 96-well plates in the presence of hIgG or OX40L blocking antibodies (oxelumab) and identified the homotypic interaction in round wells through OX40-OX40L axis is critical for high NK cell proliferation. In addition, we added oxelumab on day 3 for NK expansion culture. Blocking of the OX40/OX40L interaction in NK cells significantly reduced NK cell purity and expansion fold suggesting again that NK homotypic cell to cell interaction through OX40/OX40L is important for NK cell proliferation after the feeder cell disappeared.

In conclusion, although the characteristics of NK cells cultured with K562 and K562-OX40L were similar, K562-OX40L feeder cells triggered a significant increase of NK cell numbers by IL-2/Il-15 and short exposure to IL-21. Thus, our data suggest that not only OX40L from feeder cells is an effective co-stimulant for the preferential expansion of human NK cells, but the homotypic interaction within NK cells via the OX40-OX40L axis is another important factor to achieve highly efficient NK cell expansion. Additional work is needed to dissect the role of the OX40 and OX40L in cell signaling and cancer immunotherapy.

## Ethics Statement

Our Institutional Review Board (No. SMC 2018-02-102) approved this study and no data were used for personal identification of human PBMCs.

## Author Contributions

SoK and M-TP performed the research, analyzed data. SC, AA, and S-KK analyzed data. JK and HY contributed to NK cell expansion and RT-PCR analysis. SeK and JL generated genetically engineered K562-OX40L. JD and DC designed the research study. SoK, AA, S-HL, JD, and DC wrote the paper.

### Conflict of Interest Statement

The authors declare that the research was conducted in the absence of any commercial or financial relationships that could be construed as a potential conflict of interest.
