# Supplementary material for: Expansion of Human NK Cells Using K562 Cells Expressing OX40 Ligand and Short Exposure to IL-21
Source: Front Immunol. 2019 Apr 24;10:879. doi: 10.3389/fimmu.2019.00879 (PMC6491902; doi:10.3389/fimmu.2019.00879)
Supplement: Supplementary file 1 [file Table_1.docx]

***Supplementary Material***

***
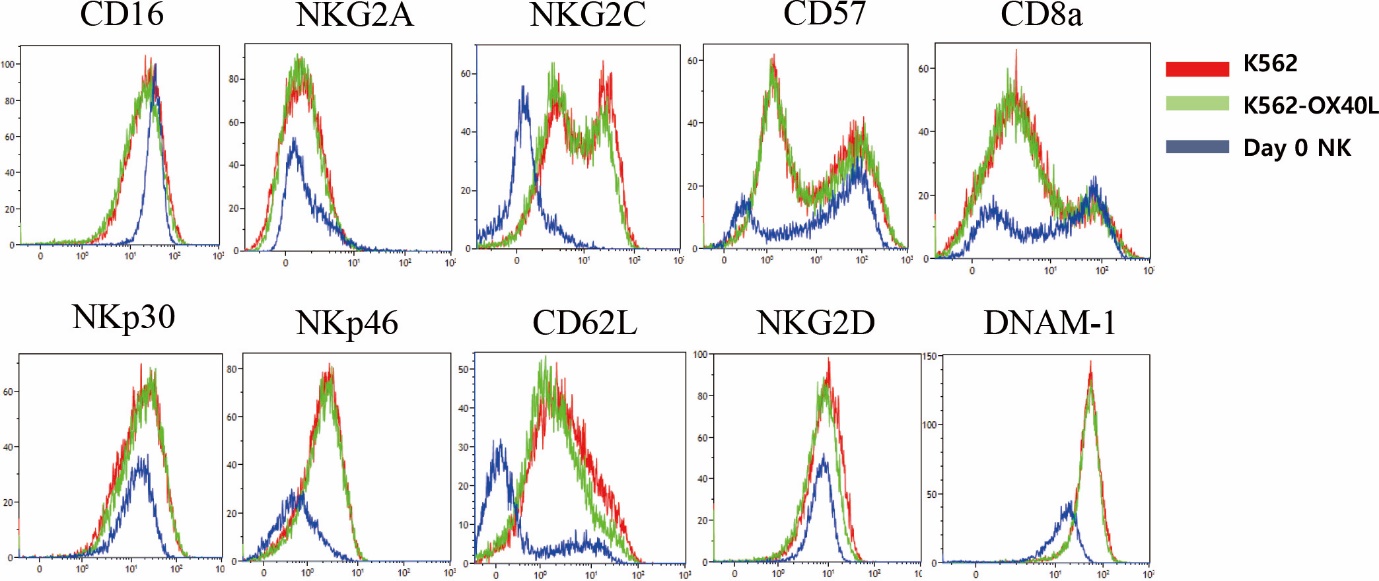
***

**Figures S1.** Expression of surface receptors on NK cells were assayed on day 7 for CD16, NKG2A, NKG2C, CD57, CD8a, NKp30, NKp46, CD62L, NKG2D and DNAM-1. Representative plots are shown in NK cell subset from the same donor, K562 group (red) and K562-OX40L group (green), NK cells on day 0 before co-culture (blue).

**
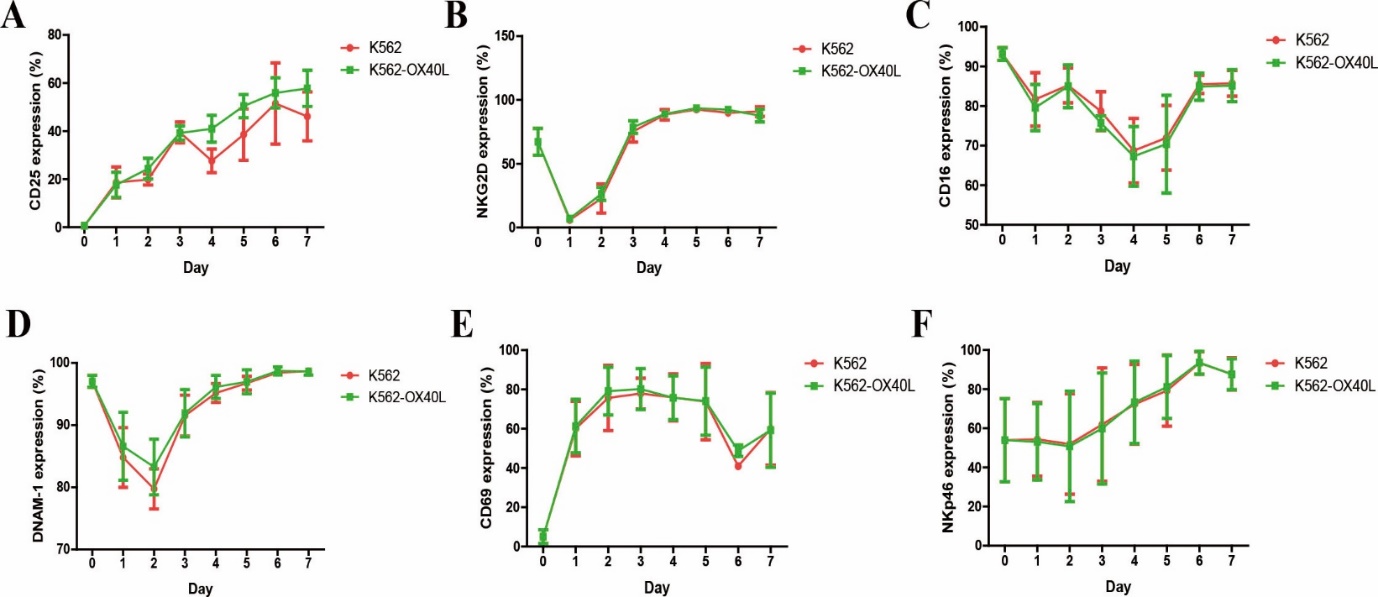
**

**Figures S2.** Surface expression of human NK cell receptors. PBMC of healthy donors were co-cultured with either K562 or K562-OX40L feeder cells. Percentage of surface expression of CD25 (A) NKG2D (B) CD16 (C) DNAM-1 (D) CD69 (E) NKp46 (F) on NK cells were examined by flow cytometry during the first week of culture (K562, n=4; and K562-OX40L, n=4; error bars, mean± SD)
