# Supplementary figures and images for: Expansion of Human NK Cells Using K562 Cells Expressing OX40 Ligand and Short Exposure to IL-21
Source: Front Immunol. 2019 Apr 24;10:879. doi: 10.3389/fimmu.2019.00879 (PMC6491902; doi:10.3389/fimmu.2019.00879)

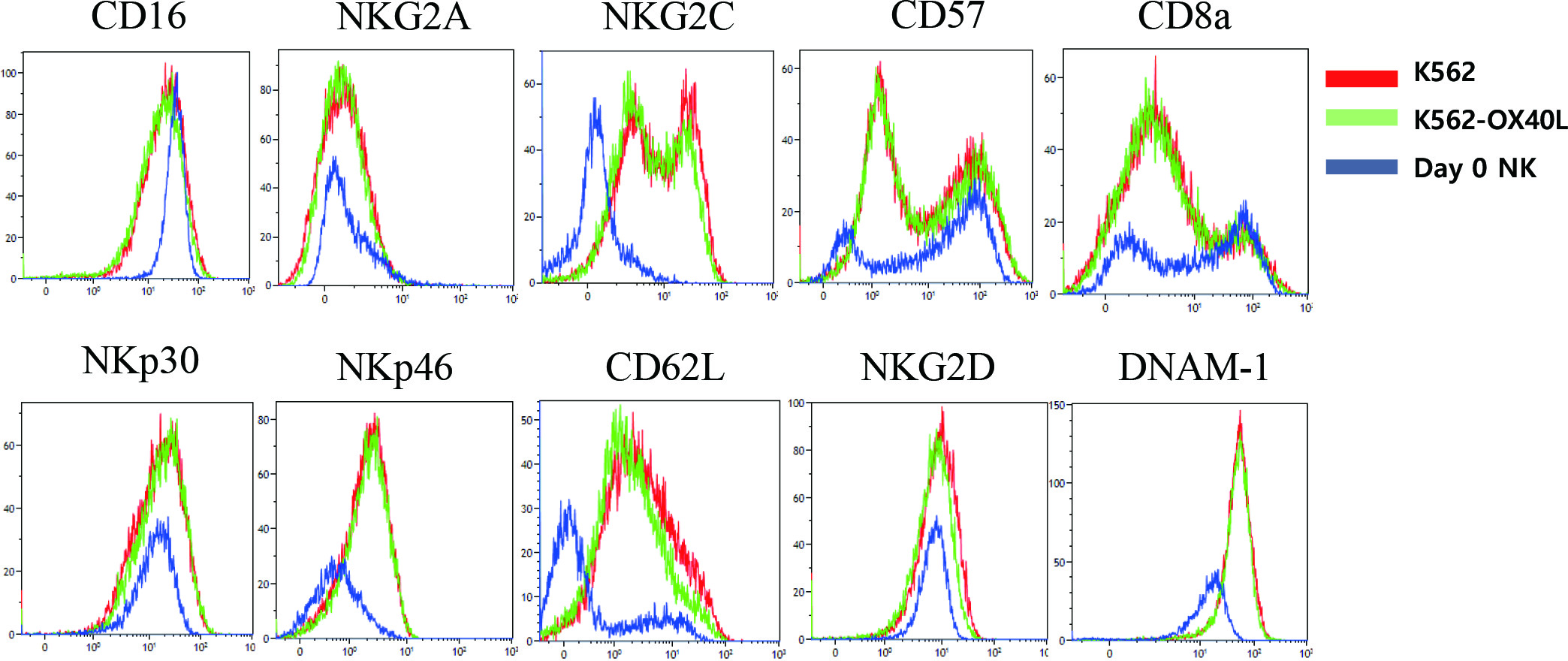

Supplement: Supplementary file 2 [file Image_1.jpeg]

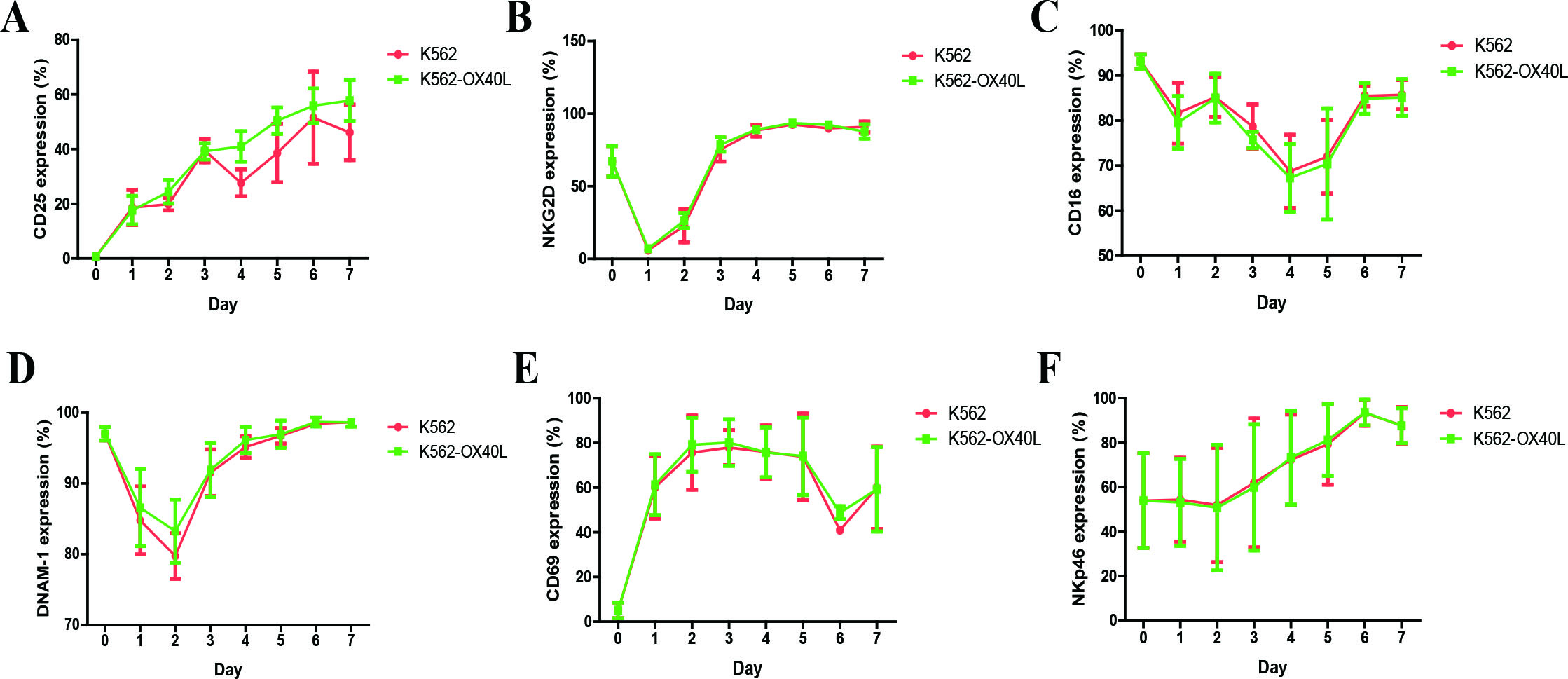

Supplement: Supplementary file 3 [file Image_2.jpeg]
